# A Multi-season Investigation of Microbial Extracellular Enzyme Activities in Two Temperate Coastal North Carolina Rivers: Evidence of Spatial but Not Seasonal Patterns

**DOI:** 10.3389/fmicb.2017.02589

**Published:** 2017-12-22

**Authors:** Avery Bullock, Kai Ziervogel, Sherif Ghobrial, Shannon Smith, Brent McKee, Carol Arnosti

**Affiliations:** Department of Marine Sciences, University of North Carolina, Chapel Hill, NC, United States

**Keywords:** enzyme activities, bacterial production, DOC, Hurricane Irene, peptidase, glucosidase, Neuse River, Tar River

## Abstract

Riverine systems are important sites for the production, transport, and transformation of organic matter. Much of the organic matter processing is carried out by heterotrophic microbial communities, whose activities may be spatially and temporally variable. In an effort to capture and evaluate some of this variability, we sampled four sites—two upstream and two downstream—at each of two North Carolina rivers (the Neuse River and the Tar-Pamlico River) ca. twelve times over a time period of 20 months from 2010 to 2012. At all of the sites and dates, we measured the activities of extracellular enzymes used to hydrolyze polysaccharides and peptides, and thus to initiate heterotrophic carbon processing. We additionally measured bacterial abundance, bacterial production, phosphatase activities, and dissolved organic carbon (DOC) concentrations. Concurrent collection of physical data (stream flow, temperature, salinity, dissolved oxygen) enabled us to explore possible connections between physiochemical parameters and microbial activities throughout this time period. The two rivers, both of which drain into Pamlico Sound, differed somewhat in microbial activities and characteristics: the Tar-Pamlico River showed higher β-glucosidase and phosphatase activities, and frequently had higher peptidase activities at the lower reaches, than the Neuse River. The lower reaches of the Neuse River, however, had much higher DOC concentrations than any site in the Tar River. Both rivers showed activities of a broad range of polysaccharide hydrolases through all stations and seasons, suggesting that the microbial communities are well-equipped to access enzymatically a broad range of substrates. Considerable temporal and spatial variability in microbial activities was evident, variability that was not closely related to factors such as temperature and season. However, Hurricane Irene's passage through North Carolina coincided with higher concentrations of DOC at the downstream sampling sites of both rivers. This DOC maximum persisted into the month following the hurricane, when it continued to stimulate bacterial protein production and phosphatase activity in the Neuse River, but not in the Tar-Pamlico River. Microbial community activities are related to a complex array of factors, whose interactions vary considerably with time and space.

## Introduction

Riverine systems are important sources of organic carbon and nutrients for coastal and estuarine systems (Paerl et al., 1998; Stow et al., 2001; Lin et al., 2007). The availability of organic matter that can be processed within rivers is dependent on multiple physical, biological, and chemical factors, including the nature and extent of allochthonous input via runoff and groundwater, as well as autochthonous production within the system (Spencer et al., 2012). The quantity and quality of organic carbon and nutrients ultimately delivered to estuaries and coasts is partially the outcome of organic matter processing by heterotrophic microbial communities within the rivers. These communities facilitate the transformation and respiration of organic matter, and regeneration of nutrients (Blackburn et al., 1996). The extent to which organic matter is processed and transformed within a riverine system is thus dependent in part on the capabilities of heterotrophic microbial communities. The initial step of organic matter transformation is typically hydrolysis via extracellular enzymes, since high molecular weight organic matter is too large to be transported directly into microbial cells. The heterotrophic microbial community therefore must utilize extracellular enzymes to hydrolyze high molecular weight organic matter to sizes sufficiently small for uptake (see Arnosti et al., 2014 for a review). Though not the sole sources of extracellular enzymes, bacterioplankton are assumed to be the major producers of extracellular enzymes in aquatic systems (Hoppe et al., 2002; Vrba et al., 2004). Only a sub-fraction of a microbial community may produce specific extracellular enzymes, but the products of hydrolysis potentially might be accessed by a wider range of organisms. The activities of extracellular enzymes may therefore benefit a wider community, and measurement of extracellular enzyme activities can represent the potential to initiate organic matter remineralization at the community-level.

A number of biological, chemical, and physical factors can influence the production of extracellular enzymes (Allison and Vitousek, 2005; Artigas et al., 2009), while the degradation of organic matter can be dependent upon such factors as substrate type (McCallister et al., 2006), availability (Sinsabaugh and Moorhead, 1994), and community nutrient demands (Rier et al., 2011). In addition, studies have shown that organic matter concentration and type change seasonally in freshwater watersheds (Singh et al., 2013). These seasonal changes are due largely to sorption of DOM on mineral soil surfaces and/or microbial breakdown of leaf litter. Singh et al. (2013) found that stormflow in summer contained DOM that was more humic in character than in spring and winter, as a result of more influence from the watershed during higher discharge periods. Therefore, changes in organic matter supply, environmental conditions, or microbial community composition across spatiotemporal scales may be reflected in the enzymatic profiles and activities of a microbial community.

Since organic matter type, concentration, and microbial community composition likely influence enzymatic activities, spatial and temporal variability of enzymatic activities could vary widely. Freshwater systems such as creeks and streams have been shown to be a medley of different microbial community activities, responding to temporally-changing environmental gradients (Frossard et al., 2012). Capturing these varying dynamics is challenging; prior studies typically have focused on sampling a range of stations over a limited timescale or on sampling a few sites over a longer period of time (e.g., Artigas et al., 2009; Millar et al., 2015).

We investigated spatial and as well as seasonal variations in microbial activities and organic matter remineralization in two distinct river systems in central and eastern North Carolina: the Neuse River and the Tar-Pamlico River. These rivers were each sampled at four different sites (two upstream sites, two downstream sites) ~12 times over a 20-month period. We carried out this extended sampling program in an effort to capture temporal variability over a range of sites. Part of this temporal variability was caused by Hurricane Irene's passage over eastern North Carolina (August 2011), an event that provided the opportunity to measure changes in microbial activities in the rivers in response to a large-scale influx of precipitation and laterally-flowing water. In order to investigate a greater range of heterotrophic capabilities, we measured the activities of enzymes capable of hydrolyzing small substrate proxies typically used to assess glucosidase and leucine aminopeptidase activities, and also used a suite of polysaccharide substrates that can measure the endo-acting activities of enzymes that cleave specific polysaccharides mid-chain. We sought to investigate the manner in which changing biological, physical, and chemical parameters may affect organic carbon cycling, as measured via activities of extracellular enzymes.

## Materials and methods

### Study sites

**Figure 1:**
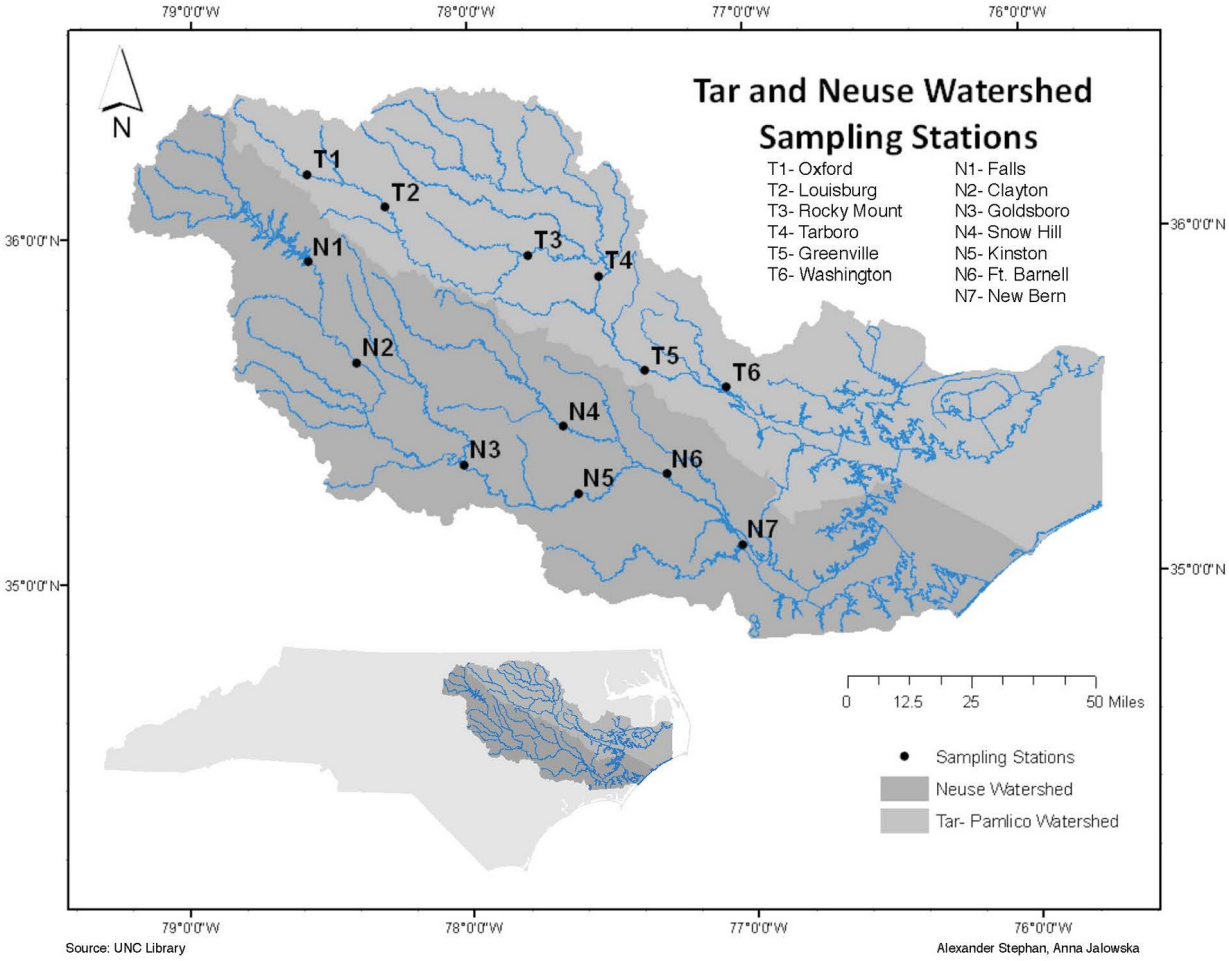
Location of sampling stations in the Tar-Pamlico and Neuse Rivers.

The Neuse and Tar-Pamlico Rivers, extending from central to eastern North Carolina, feed into Pamlico Sound (Paerl et al., 2010; Figure 1). The Albemarle-Pamlico Sound estuary system is the second largest estuary system in the United States (Paerl et al., 2010), and provides significant nursery area for commercially-important fisheries on the U.S. Atlantic coast (Burkholder et al., 2006). Although the Albemarle-Pamlico Sound, as well as other estuary systems, serves as an important link between terrestrial/riverine systems and the marine environment (Paerl et al., 1998), the dynamics of organic matter processing occurring in the Neuse and Tar-Pamlico Rivers are not well-studied.

The two river systems have contrasting watersheds (Burgess, 2013a,b). The Neuse River is heavily urbanized upstream, with a population of over 1.5 million residing within its watershed (Burgess, 2013a), and is also subject to heavy industrialized agricultural use. The Tar-Pamlico River is a smaller, less developed river both in terms of agricultural and urban development, but it is the largest tributary of the Pamlico River Estuary (Overton et al., 2012; Table 1). The mean discharge to the Albemarle-Pamlico estuarine system is 190 m^3^ s^−1^ for the Neuse River and 148 m^3^ s^−1^ for the Tar-Pamlico River (Lin et al., 2007). Downstream salinities also differ between rivers: the Tar-Pamlico endmember salinities are close to zero (compared to the Neuse) because the Tar-Pamlico estuary is relatively narrow and the influence of ocean waters due to wind mixing is much more limited relative to that in the Neuse estuary, which has a more open morphology.

**Table 1:** Characteristics of the Neuse and Tar-Pamlico Rivers (O'Driscoll et al., 2010).

	**Neuse river**	**Tar-Pamlico river**
Maximum elevation (m)	183	218
River length (km)	443	346
Basin area (km^2^)	15,700	15,923
Population	1,687,462	472,629
Land use (%)		
Forested	30	36
Grassland	4	3
Agriculture	26	22
Urban	15	5
Wetland	19	18

Surface water samples were collected from four stations in each river: N1, N2, N6, and N7 in the Neuse River, and T1, T2, T5, and T6 in the Tar River (Figure 1). These stations were chosen to include the different land-use impacts of the rivers (urbanization of upstream stations; agricultural effects on downstream stations), as well as the transition from a freshwater to an estuarine ecosystem. Collection dates (Supplementary Table 1) varied slightly among sites, due to the complex logistics of sampling eight different locations spread across a considerable distance. Due to the timing of our study, we also partially captured the influence of a major storm event (Hurricane Irene) on the lower stations of the Tar-Pamlico and Neuse Rivers in August 2011.

### Sample collection

Surface water samples were collected over a 20-month period (November 2010 to June 2012) from each of the four stations in the Neuse and Tar-Pamlico Rivers (Figure 1). Samples were collected in 33 L Nalgene carboys and stored at *in-situ* temperatures during transportation back to UNC-Chapel Hill. Measurements of enzyme activities and bacterial productivity were initiated upon return to the lab, and samples for cell counts were preserved (see below). Dissolved oxygen, temperature, salinity, and pH data were collected on site using a YSI (YSI Inc. 556MPS) (Supplementary Table 1). River discharge and gage height for most stations was obtained from the USGS's monitoring website (http://waterdata.usgs.gov/nc/nwis/rt). Note that the USGS does not have a monitoring site at T6, and USGS data collection was ended at N7 in 2009, so for the downstream stations we used gage height (Supplementary Figure 1) and discharge data from T4 and T5 for the Tar River, and N5 and N6 for the Neuse River (Figure 1; Supplementary Table 4). Hurricane Irene struck eastern North Carolina on 27–28 August 2011. Post-hurricane sampling of the Tar-Pamlico River occurred at Stns. 5 and 6 on August 29, 2011. Somewhat later post-hurricane sampling was carried out at Stns. T5 and T6 and Stn. N7 on Sept. 14th (Stns. T1 and T2 and Stns. N1 and N2 were sampled on Sept. 12th); all sampling dates are shown in Supplementary Table 1.

### Extracellular enzyme activities

Activities of exo-acting (terminal-unit cleaving) as well as endo-acting (mid-chain cleaving) enzymes were measured using two different methods. Low molecular weight substrate proxies [4-methylumbelliferone- (MUF-) and 4-methylcoumarinyl-7-amide- (MCA-) labeled substrates] were used to measure α- and β-glucosidase, leucine aminopeptidase, and phosphatase activities, after the method of Hoppe (1983; Hoppe et al., 1988). Triplicate water samples from each station were amended with substrate proxies to a final concentration of 400 μM (this concentration was chosen at the start of the project, from a saturation curve made to determine the appropriate saturation concentration of each substrate in the river water). Killed controls consisted of autoclaved water to which substrate was added. Samples were incubated for a period of 3–5 h at *in situ* or near *in-situ* temperature; an initial time-zero measurement was taken at the start of this period, and two to three subsequent time points were measured during this period. For each measurement, a 1-ml aliquot was taken from the incubating sample and combined with 1 ml of 20 mM borate buffer, and fluorescence was measured using single-cell fluorometers (Turner Biosystem TBS-380 or a Promega Quantifluor-ST). A dilution curve was made with each fluorophore in autoclaved river water to determine a fluorescence-hydrolysis rate conversion factor for each river. Hydrolysis rates were then calculated using the conversion factors and fluorescence measurements.

The activities of extracellular enzymes responsible for endo-acting (mid-chain cleaving) hydrolysis of a specific set of polysaccharides were measured using six distinct fluorescently labeled (FLA) polysaccharides (Arnosti, 1996, 2003). Arabinogalactan, chondroitin, fucoidan, laminarin, pullulan, and xylan (all obtained from Sigma-Aldrich USA) were labeled with fluoresceinamine as described in Arnosti (2003). These polysaccharides were selected because they are derived from a range of terrestrial (xylan, arabinogalactan) and marine (laminarin, xylan, fucoidan, pullulan, chondroitin) sources, and/or enzymes hydrolyzing these polysaccharides have been identified in marine bacteria and in marine bacterial genomes (for details, see e.g., Bold, 1985; Arnosti, 2000; Alderkamp et al., 2007; Wegner et al., 2013). In addition, the activities of enzymes hydrolyzing all of these polysaccharides have been measured in marine (Arnosti et al., 2011) as well as freshwater systems (Ziervogel et al., 2014). Because of the time and resources required for measurements with FLA-polysaccharides, polysaccharide hydrolysis rates were measured in duplicate, only at the upriver-most and downriver-most station in each river. At these stations (Stns. T1, T6; N1, and N7), duplicate live water samples, as well as an autoclaved control water sample for each station, were separately amended with one of each of the six substrates to a final concentration of 3.5 nM monosaccharide equivalent. A time-zero measurement was immediately taken, and the samples were then incubated in the dark at near *in-situ* temperature, with subsamples withdrawn periodically. After processing the samples, we found that, with very few exceptions, all polysaccharides were hydrolyzed at 3 days, the first time-point after the zero-time sample, so the data reported are all from this time point. Inconsistencies in sampling timepoints after 3 days in any case preclude use of later timepoints across the dataset.

Samples for measurement of polysaccharide hydrolase activities were collected by filtering 1–3 ml of sample water through a 0.2 μm cellulose acetate-membrane + GF-prefilter syringe filter (Sartorius Stedim Biotech, Germany), and freezing samples at −20°C until analysis. Hydrolysis was measured via changes in the molecular weight distribution of the FLA-labeled polysaccharide using gel permeation chromatography, as described in detail in Arnosti (2003). Several samples were lost prior to analysis from the Stn. T1 sample set: (date/substrate): 01/11 (fucoidan), 04/11 (pullulan), 06/11 (fucoidan), and 06/12 (xylan). At Stn. T6, missing samples were as follows: 09/11 (fucoidan), 11/11 (fucoidan), and 06/12 (laminarin).

### Bacterial cell counts and production

Aliquots of water were fixed for bacterial cell counts, following Porter and Feig (1980). Staining was carried out with 4′, 6-diamidino-2-phenylindole (Sigma-Aldrich USA), and slides were counted under an epifluorescence microscope (Olympus U-RFL, Olympus USA) using MetaMorph Microscopy software (Molecular Devices USA). 10 fields of view were counted per slide, with duplicate slides made for each river station.

Bacterial production was measured using ^3^H-leucine incorporation (Kirchman, 2001). These measurements were only initiated in January 2011, so no data are available for samples collected during November/December 2010. Water from the upstream- and downstream-most stations (the same stations used to measure polysaccharide hydrolysis) in each river, plus autoclaved control water, was amended with ^3^H-leucine to a final concentration of 20 nM. Samples were incubated for 1–2 h; following this incubation period, reactions were terminated using 100% trichloroacetic acid (TCA). Samples were then concentrated and washed with 80% ethanol before drying over night. Samples were then amended with scintillation liquid and allowed to sit for a 2-day period before analysis in a scintillation counter (Perkin Elmer TriCarb 3110 TR).

### Dissolved organic carbon

Water samples from each station were filtered through 0.2 μm cellulose acetate-membrane + GF-prefilter syringe filter (Sartorius Stedim Biotech, Germany) into pre-combusted glass scintillation vials and frozen at −20°C until further analysis. Dissolved organic carbon concentrations from these samples was measured via high temperature catalytic oxidation and non-dispersive infrared detection on a Shimazdu TOC-L series instrument (Shimadzu Corp. Kyoto). Samples were acidified to a pH < 2 and sparged with commercially obtained CO_2_ free, zero-grade air for 10 min for inorganic carbon removal. Standards were generated from dilution of commercially prepared potassium hydrogen phthalate [KHP] (La-Mar-Ka Inc., Baton Rouge, LA) with 18.2 MΩ ultrapure water.

### Statistical analyses

Environmental data and microbial activity measurements described above were analyzed to look for correlations using the corrplot package in R (R Core Team, 2014), run in R version 3.3.3. The same package was used to look for correlations among activities of individual polysaccharide hydrolases. *T*-test analyses of potential effects of season, station, and the effects of Hurricane Irene on microbial activity measurements were also analyzed using R.

## Results

### Environmental and hydrological characteristics

**Figure 2:**
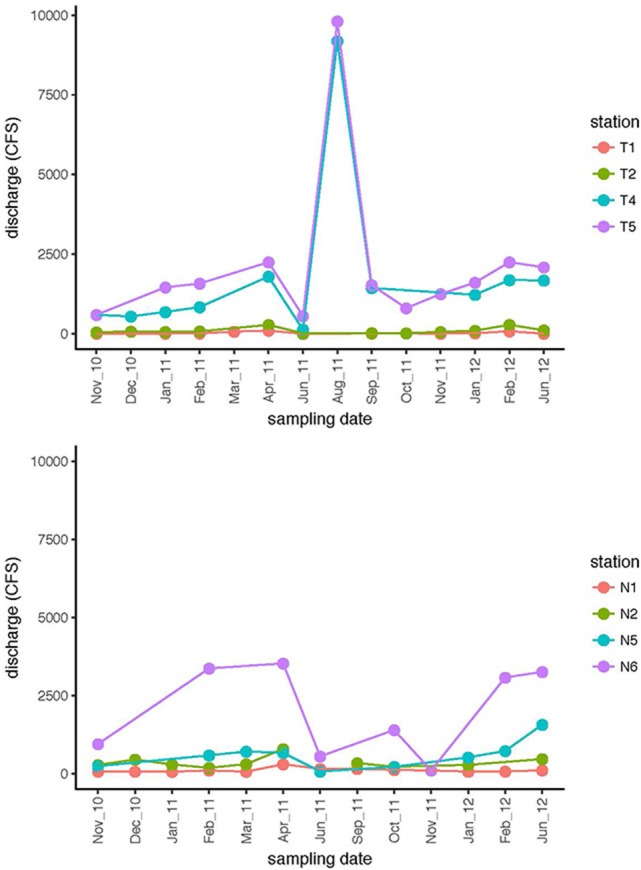
Discharge (cubic feet per second) at four stations in the Tar-Pamlico River and the Neuse River on dates when samples were collected. Filled circles are from the dates at which samples were collected; lines connect consecutive sampling dates.

Environmental data including dissolved oxygen (DO), pH, and salinity (Supplementary Table 1), as well as river discharge (Figure 2), were used establish a picture of the seasonal environmental characteristics and dynamics of both rivers, and to investigate potential connections between community activity and river characteristics. River discharge indicated that the upstream and downstream stations were hydrologically decoupled (Figure 2). Discharge volume for Stns. T1 and T2 tracked together, and were distinctly separated from discharge volume at Stns. T4 and T5. Discharge data from the Neuse River likewise showed decoupling of upstream (Stns. N1 and N2) stations from the downstream-most station for which discharge data are available (Stn. N6). These patterns were supported by other chemical and physical data (Supplementary Table 1): in the Neuse River, salinity remained near zero upstream (Stns. N1, N2); the downstream stations exhibited greater fluctuations in salinity, varying between freshwater and estuarine conditions. Salinity of the Tar-Pamlico River, however, remained close to zero, even at the station farthest downriver (Stn. T6; Supplementary Table 1). Temperature ranges changed seasonally in both rivers, with the annual variations in the Neuse River between 4 and 30°C, and near 0 to 29°C in the Tar-Pamlico River. At each sampling point, temperatures were broadly comparable among stations, although downriver stations were frequently slightly warmer than upriver stations, and the temperature difference between Stns. T1 and T6 was typically a few degrees greater than between Stns. N1 and N7 (Supplementary Table 1). Dissolved oxygen (DO) followed an inverse relationship with temperature in both rivers. The range of DO for both downstream stations was greater than the DO ranges upstream.

**Figure 3:**
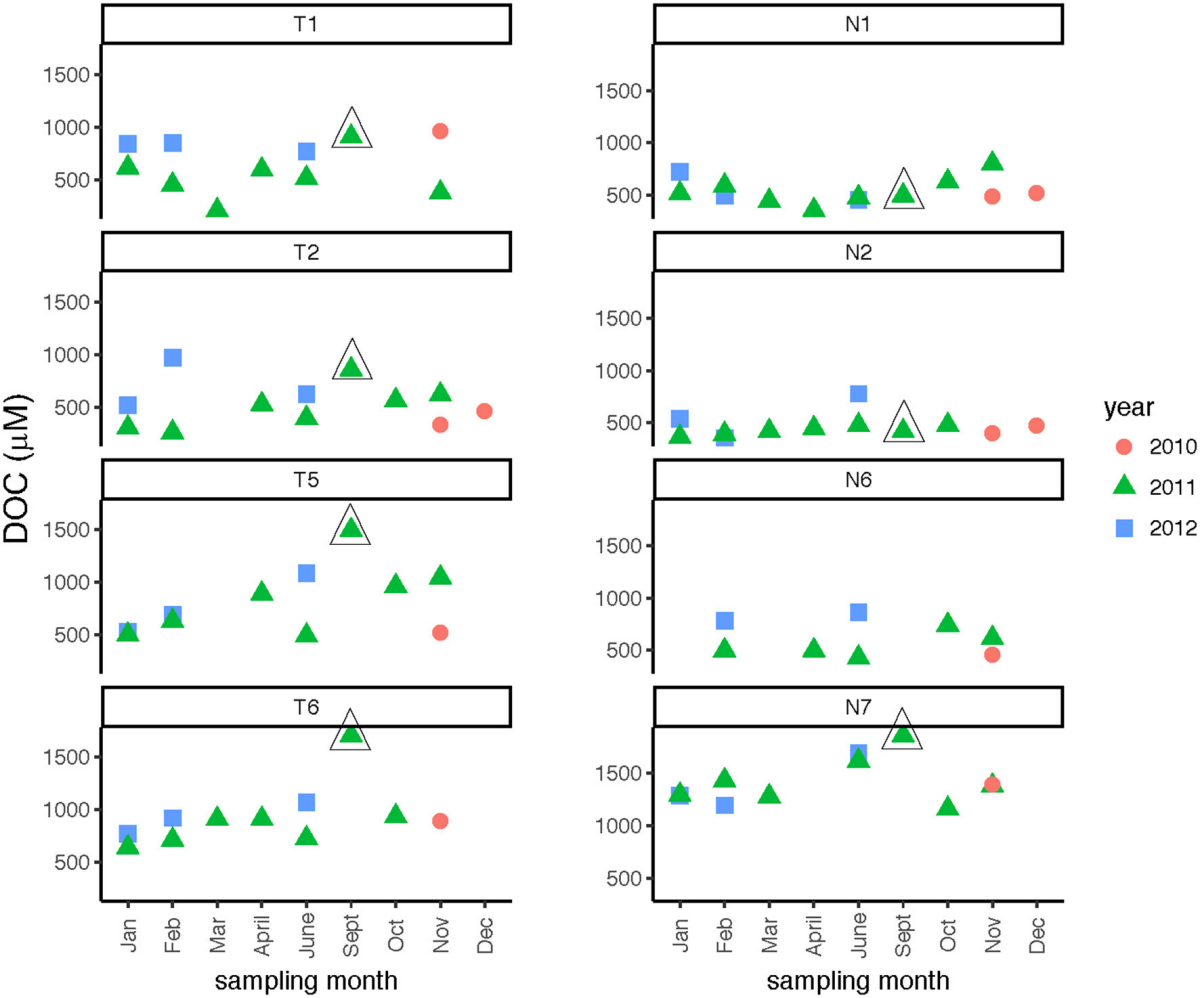
Dissolved organic carbon concentrations at four stations each in the Tar-Pamlico and Neuse River. “T” corresponds to Tar-Pamlico stations, “N” corresponds to Neuse stations. Symbols outlined with triangles show sampling carried out in the month post Hurricane Irene.

DOC concentrations showed broad patterns across spatial and temporal scales. In the Neuse River, DOC was consistently highest (near or above 1,200 μmol C L^−1^) at the downstream-most station, Stn. N7 (Figure 3; Table 2), while DOC concentrations at Stns. N1, N2, and N6 ranged from ~400 to 800 μmol C L^−1^. Stn. N2 exhibited the least temporal variation in concentration. No seasonal trends were evident, but the highest DOC measured at Stn. N7 (1,861 μmol C L^−1^) was in September 2011, following Hurricane Irene in August 2011. In the Tar-Pamlico River, DOC concentrations at most stations and seasons exhibited wider temporal variability than in the Neuse River, ranging from ca. 300 to 1,000 μmol C L^−1^. There was no distinct seasonal trend (Figure 3; Table 2), but DOC concentrations were higher downstream than upstream (Table 2), and the highest DOC concentrations (exceeding 1,700 μmol C L^−1^) were also recorded at the downriver stations, Stns. T5 and T6, in September 2011, following Hurricane Irene (Figure 3).

**Table 2:** *P*-values (*T*-tests) to determine statistical significance (as shown by *P* < 0.05; bold font) of microbial activities, cell counts, and DOC concentration by location and time.

	**β-glu**	**α-glu**	**Leu**	**Phosph**.	**Sum FLA**	**DOC**	**Bact prod**	**Cell counts**
Jan/Feb vs. other months	**8.33E-04**	**9.96E-03**	**6.76E-04**	**8.18E-08**	**1.60E-16**	0.341	**3.44E-07**	0.867
Tar/Neuse	**0.004**	0.051	0.187	**0.009**	0.937	0.738	0.554	0.446
Upriver/downriver	0.642	0.537	0.056	0.177	0.937	**4.31E-08**	**0.033**	0.440
Tar: T6 vs. other Tar stns	0.752	0.411	**0.049**	0.936	0.937	**0.014**	0.819	
Neuse: N7 vs. other Neuse stns	0.860	0.343	0.089	0.572	0.184	**0.001**	**0.014**	0.965
T6/N7 differences	0.089	0.300	0.374	0.089	0.432	**2.57E-04**	0.091	0.577
Hurricane Irene	0.090	0.435	0.292	**0.001**	0.057	**0.006**	0.488	**0.017**

*β-glu, β-glucosidase activities; α- glu, α-glucosidase activities; Leu, leucine aminopeptidase activities; phosph, phosphatase activities; sum FLA, summed polysaccharide hydrolase activities; bact prod, bacterial protein production; DOC, dissolved organic carbon concentration. Hurricane Irene-affected times and locations were defined as Stations T5/T6 and N6/N7 in Sept 2011 (and for T5/T6, also in Aug 2011) compared to all other months at the same stations*.

### Microbial cell counts and leucine incorporation

**Figure 4:**
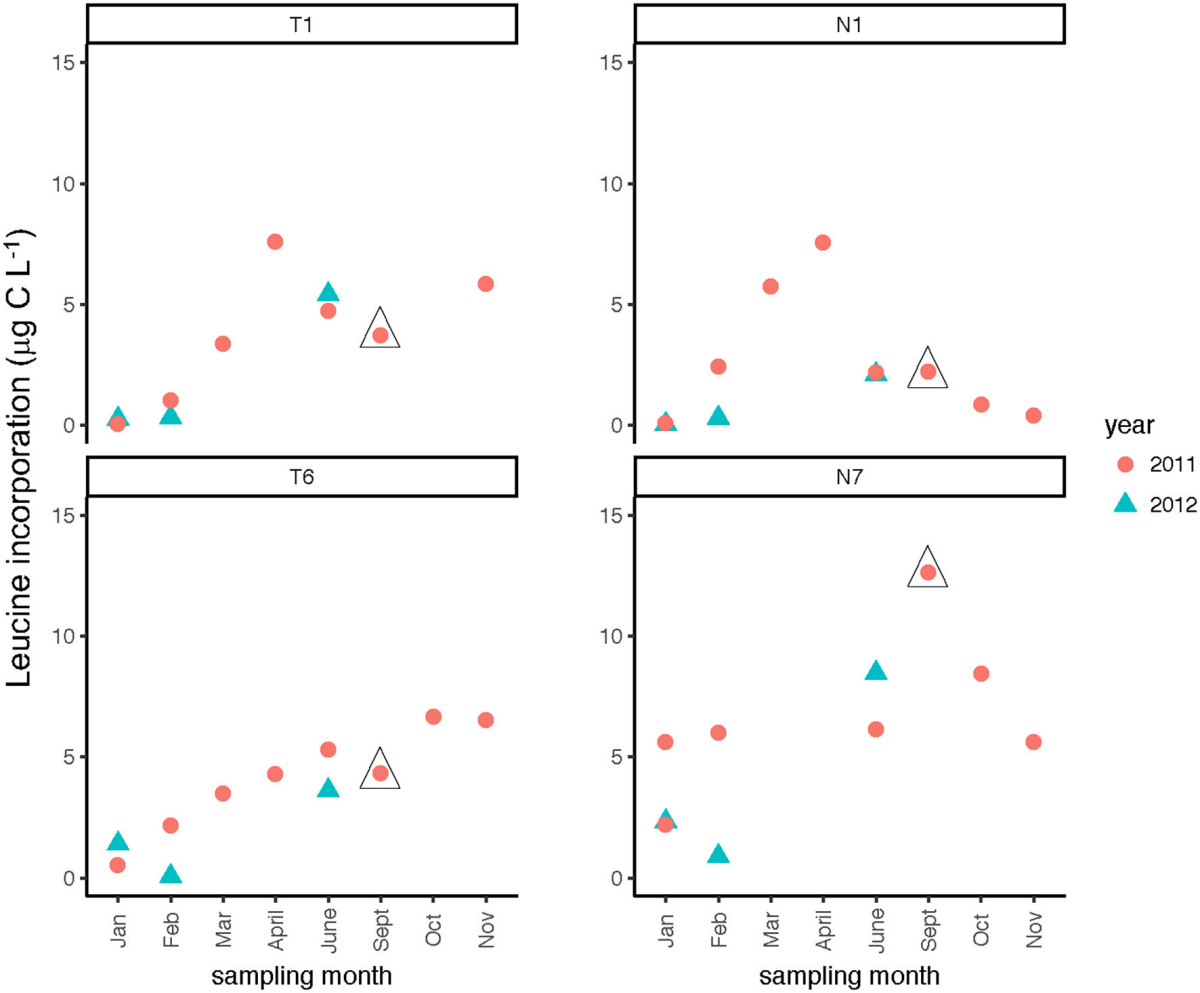
Bacterial protein production measured at two stations each in the Tar-Pamlico and Neuse River. “T” corresponds to Tar-Pamlico stations, “N” corresponds to Neuse stations. Symbols outlined with triangles show sampling carried out in the month post Hurricane Irene.

Seasonally, bacterial numbers were highest in the late winter and early spring (Feb.–April) of 2011 (*t*-test; *p* < 0.01). In both rivers, months sampled in the winter and late spring of 2012 had lower bacterial abundance than their 2011 counterparts. Bacterial abundance (Supplementary Table 2) varied by a factor of 10 over the time course of the study. Bacterial protein production (Figure 4) showed slight increases during the spring through fall months, with minima occurring during the winter months (Jan/Feb) for both rivers (Table 2). When normalized on a per-cell basis (Supplementary Table 2), the summer and late fall months showed highest bacterial production. For most months, bacterial protein production rates normalized to cell abundance were higher downstream (Stns. T6, N7) compared to upstream (Stns. T1, N1) in both rivers (Supplementary Table 2). The highest bacterial protein production measured during the study (per cell, as well as on a volume basis) was measured in the Neuse River at Stn. N7 in Sept. 2011, after the passage of Hurricane Irene (Figure 4).

### Activities of glucosidase, peptidase, and phosphatase enzymes

**Figure 5:**
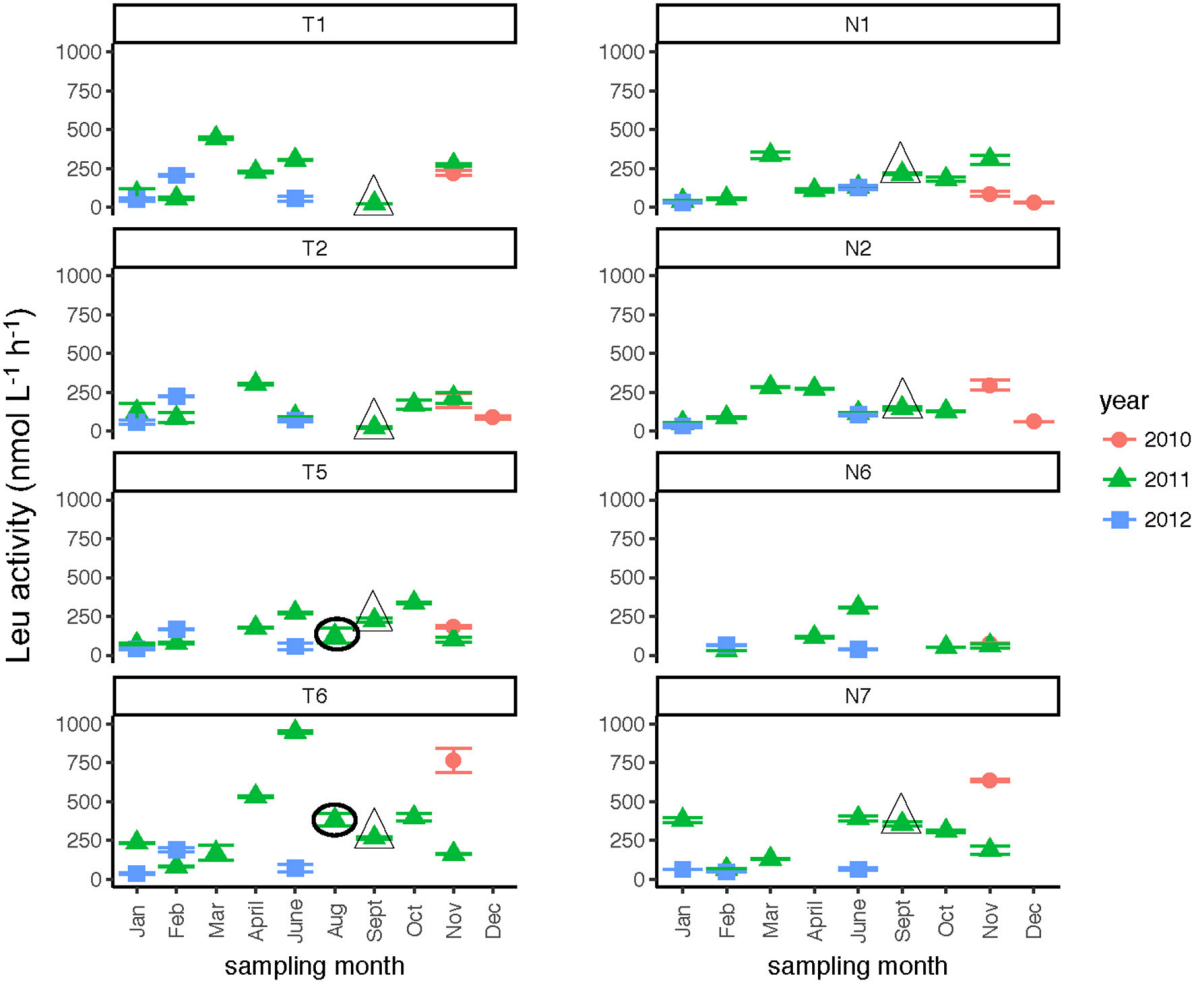
Leu-MCA activities (average and standard deviations) at four stations in the Neuse and Tar-Pamlico Rivers from 2010 to 2012. “T” corresponds to Tar-Pamlico stations, “N” corresponds to Neuse stations. Symbols outlined with triangles show sampling carried out in the month post Hurricane Irene. Circled symbols (Stns. T5 and T6 only) show samples collected in August 2011, shortly after Hurricane Irene crossed through eastern North Carolina.

Leucine amino peptidase (Leu-MCA), glucosidase (α- and β-glu), and phosphatase activities were measured immediately upon return of the samples to the lab to assess microbial heterotrophic activities across locations and seasons. Leu-MCA hydrolysis rates were highest at the downstream-most station for the Tar-Pamlico River (Figure 5; Table 2). Averaged across all timepoints, Leu-MCA hydrolysis rates were 182, 147, 156 nmol L^−1^ h^−1^ for Stns. T1, T2, and T5, respectively, and approximately double-−324 nmol L^−1^ h^−1^–at Stn. T6. For the Neuse River, averaged across all timepoints, Leu-MCA hydrolysis was 137, 144, and 93 nmol L^−1^ h^−1^ at Stns. N1, N2, and N6, respectively, and considerably higher (239 nmol L^−1^ h^−1^) for Stn. N7. Although in both cases minimum rates occurred during winter months (Table 2), there were no overall seasonal trends for either river.

**Figure 6:**
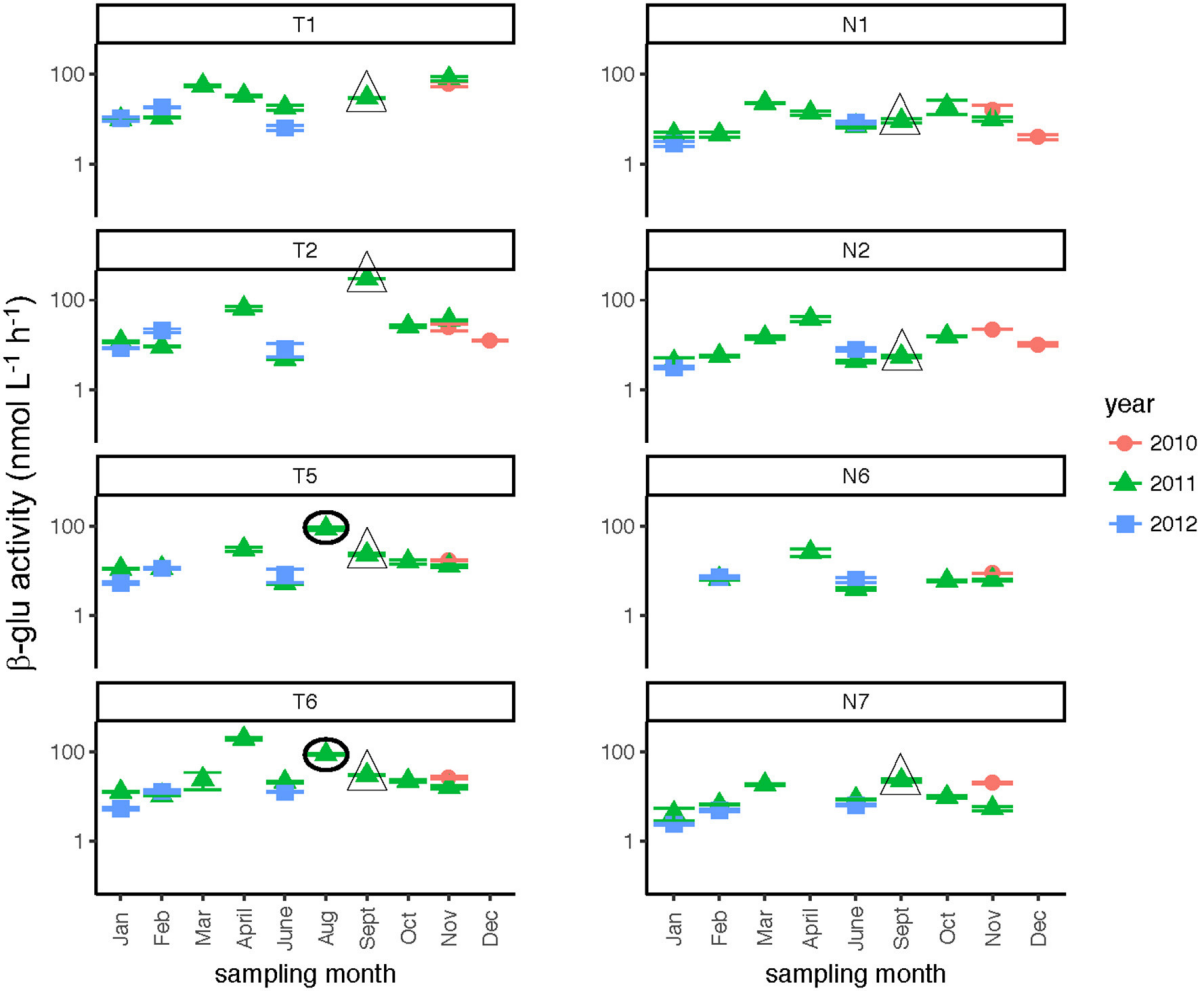
β-glucosidase activities (average and standard deviations) at four stations each in the Neuse and Tar-Pamlico Rivers from 2010 to 2012. “T” corresponds to Tar-Pamlico stations, “N” corresponds to Neuse stations. Symbols outlined with triangles show sampling carried out in the month post Hurricane Irene. Circled symbols (Stns. T5 and T6 only) show samples collected in August 2011, shortly after Hurricane Irene crossed through eastern North Carolina. y-axis is a logarithmic scale.

**Figure 7:**
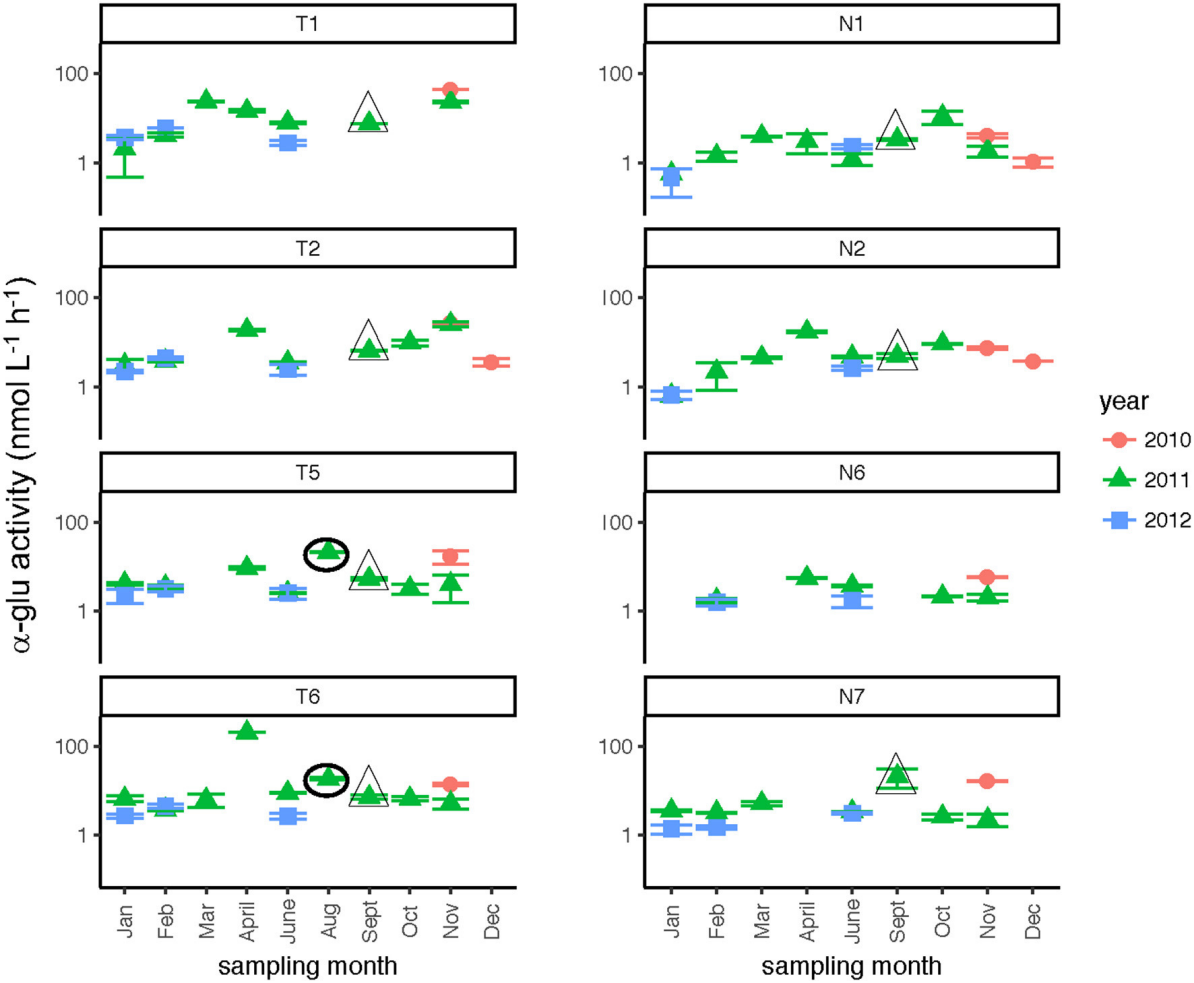
α-glucosidase activities (average and standard deviations) at four stations each in the Neuse and Tar-Pamlico Rivers from 2010 to 2012. “T” corresponds to Tar-Pamlico stations, “N” corresponds to Neuse stations. Symbols outlined with triangles show sampling carried out in the month post Hurricane Irene. Circled symbols (Stns. T5 and T6 only) show samples collected in August 2011, shortly after Hurricane Irene crossed through eastern North Carolina. y-axis is a logarithmic scale.

Glucosidase hydrolysis rates were generally higher in the Tar-Pamlico than the Neuse River, although this difference was statistically significant only for β-glu activities (Table 2). Glucosidase hydrolysis rates averaged close to 30 nmol L^−1^ h^−1^ in the Tar-Pamlico River and ~10 nmol L^−1^ h^−1^ in the Neuse River. In both rivers, β-glu activities were generally a factor of 2–3 higher than α-glu activities (Figures 6, 7; Supplementary Table 5); β-glu activities also showed a greater dynamic range (difference between lowest and highest rates). In both rivers, glucosidase activities were lowest in January and February (Table 2), but otherwise varied considerably by month and station.

**Figure 8:**
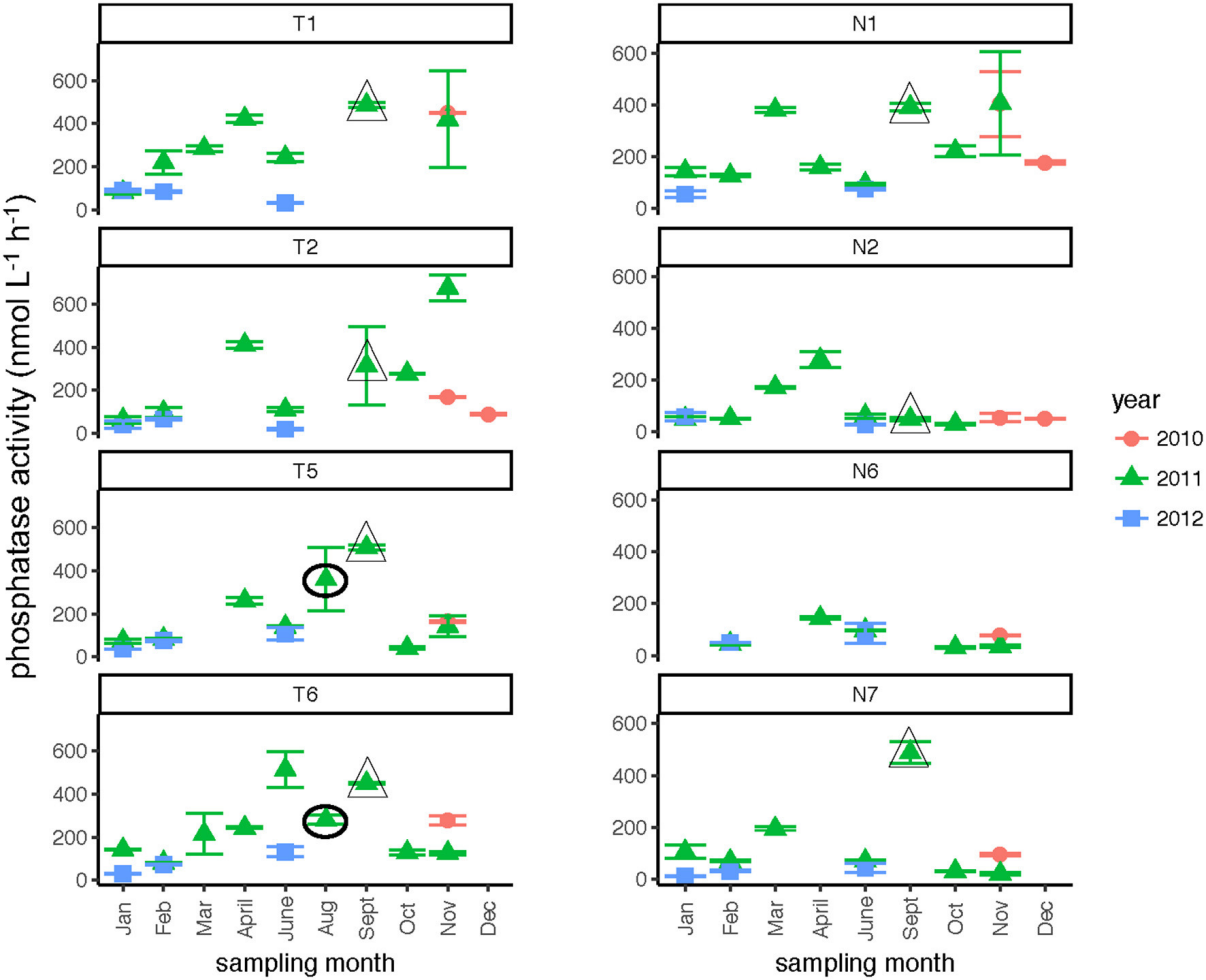
Phosphatase activities (average and standard deviations) at four stations each in the Neuse and Tar-Pamlico Rivers from 2010 to 2012. “T” corresponds to Tar-Pamlico stations, “N” corresponds to Neuse stations. Symbols outlined with triangles show sampling carried out in the month post Hurricane Irene. Circled symbols (Stns. T5 and T6 only) show samples collected in August 2011, shortly after Hurricane Irene crossed through eastern North Carolina.

Phosphatase activities typically were higher in the Tar-Pamlico than in the Neuse River (Figure 8; Table 2). In both rivers, phosphatase activities were typically low in January and February, and were considerably higher during other months of the year (Table 2). Activities did not differ systematically between upstream and downstream locations (Table 2). At Stn. N7, however, a notably high maximum (ca. 500 nmol L^−1^ h^−1^) was measured in Sept. 2011, the month after Hurricane Irene, when bacterial productivity and the DOC concentration at this station also reached maxima (Figures 3, 4).

### Activities of polysaccharide-hydrolyzing enzymes

**Figure 9:**
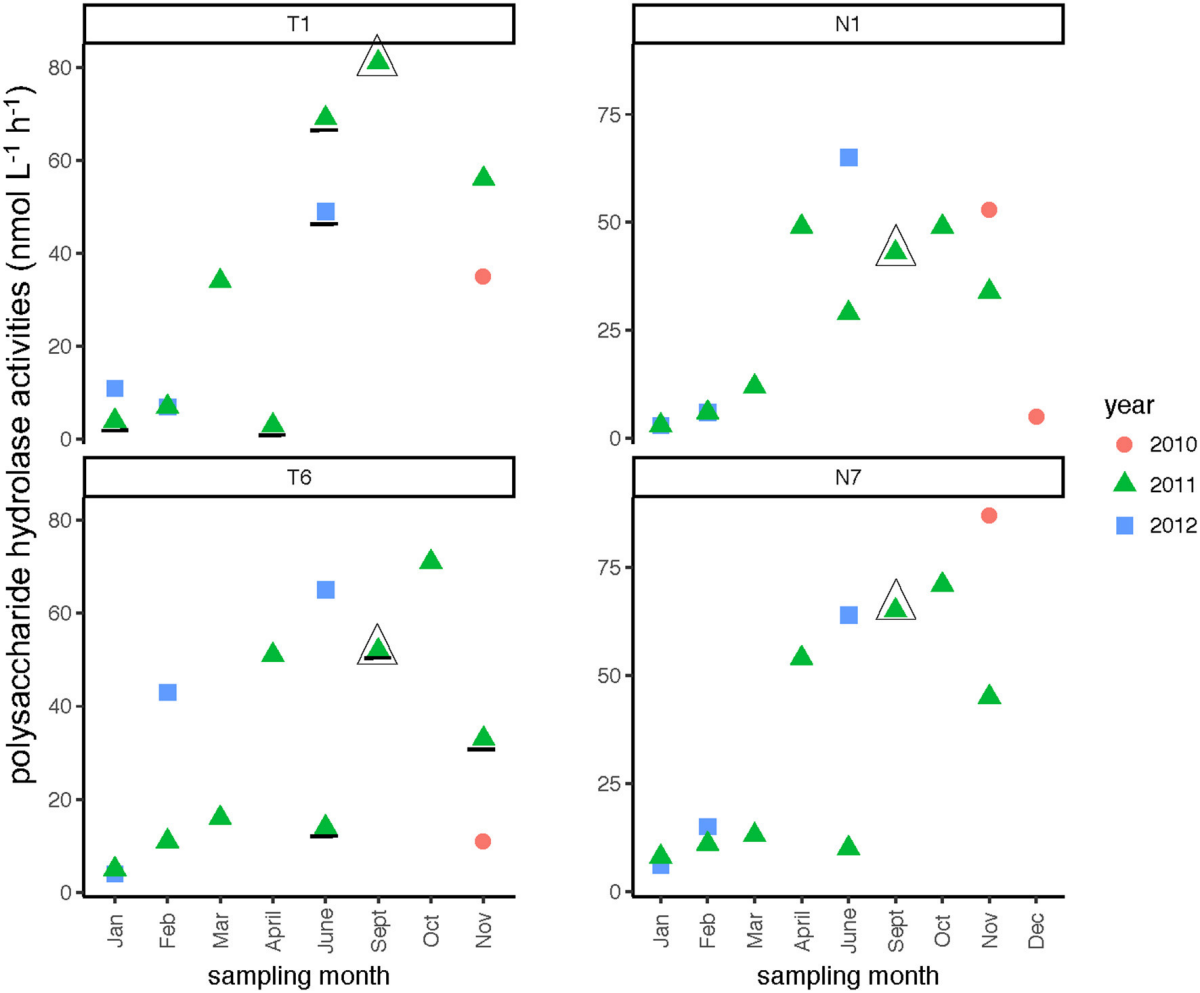
Summed polysaccharide hydrolase activities in the Neuse and Tar-Pamlico Rivers from 2010 to 2012. “T” corresponds to Tar-Pamlico stations, “N” corresponds to Neuse stations. Plot shows the average of summed activities; note that hydrolysis of each individual polysaccharide was measured in duplicate. Symbols that are underlined for Stns. T1 and T6 indicate dates for which specific polysaccharide hydrolase samples were missing (see Methods). All polysaccharide hydrolase data are shown in Supplementary Table 3.

In both rivers, a broad spectrum of polysaccharide hydrolase activities was measured, with all six polysaccharides hydrolyzed at many sampling dates and stations (Supplementary Table 3). Summed polysaccharide hydrolysis rates were frequently lowest in the winter months (Table 2), but otherwise varied considerably (Figure 9). The relative contribution of each polysaccharide hydrolase activity to summed activities was quite dissimilar, however. Chondroitin and xylan hydrolysis together averaged 58–65% of the total contributions to the summed polysaccharide hydrolysis rates across all seasons and stations in both rivers, irrespective of whether summed activities were high or low (Supplementary Table 3). For most stations, hydrolysis rates generally decreased in the order xylan, chondroitin >> laminarin > arabinogalactan with smaller contributions from fucoidan and pullulan (Supplementary Table 3). The annual range of summed hydrolysis rates was similar among all stations, from 3 to 65 nmol monomer L^−1^ h^−1^ at Stn. N1, 6–87 nmol monomer L^−1^ h^−1^ at Stn. N7, 3–81 nmol monomer L^−1^ h^−1^ at Stn. T1, and 4–71 nmol monomer L^−1^ h^−1^ at Stn. T6 (Figure 9).

### Correlations among microbial activities and environmental parameters

**Figure 10:**
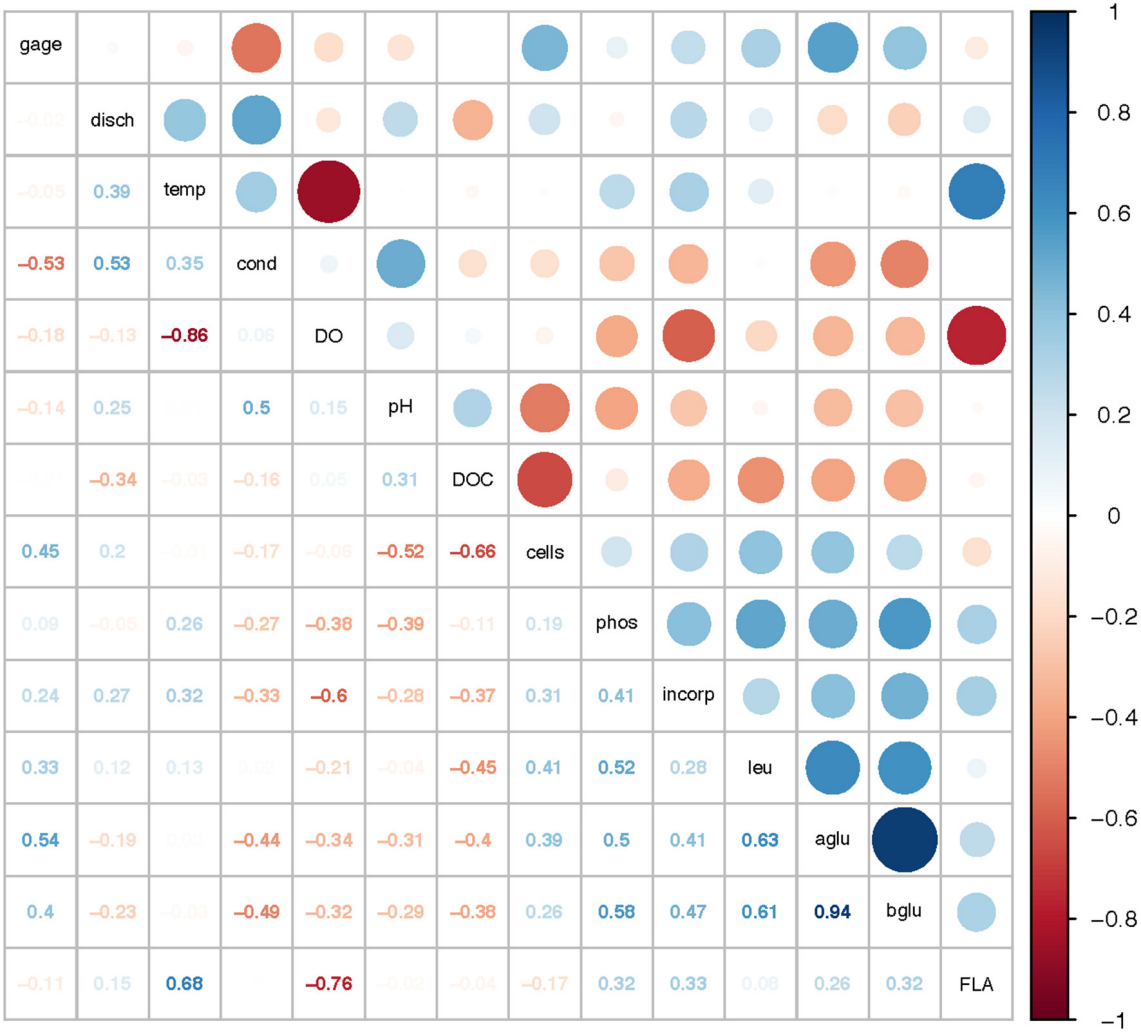
Correlation plot for physical/chemical and microbial activity data. Gage, gage height; disch, discharge; temp, temperature; cond, conductivity; DO, dissolved oxygen; pH, pH; DOC, dissolved organic carbon; cells, cell counts; phos, phosphatase activities; incorp, bacterial protein production (leucine incorporation); leu, leucine-aminopeptidase (Leu-MCA); aglu, a-glucosidase; bglu, b-glucosidase; FLA, summed polysaccharide hydrolase activities. Colors show intensity of positive (blue) and negative (red) correlations; numbers show corresponding correlation coefficients.

**Figure 11:**
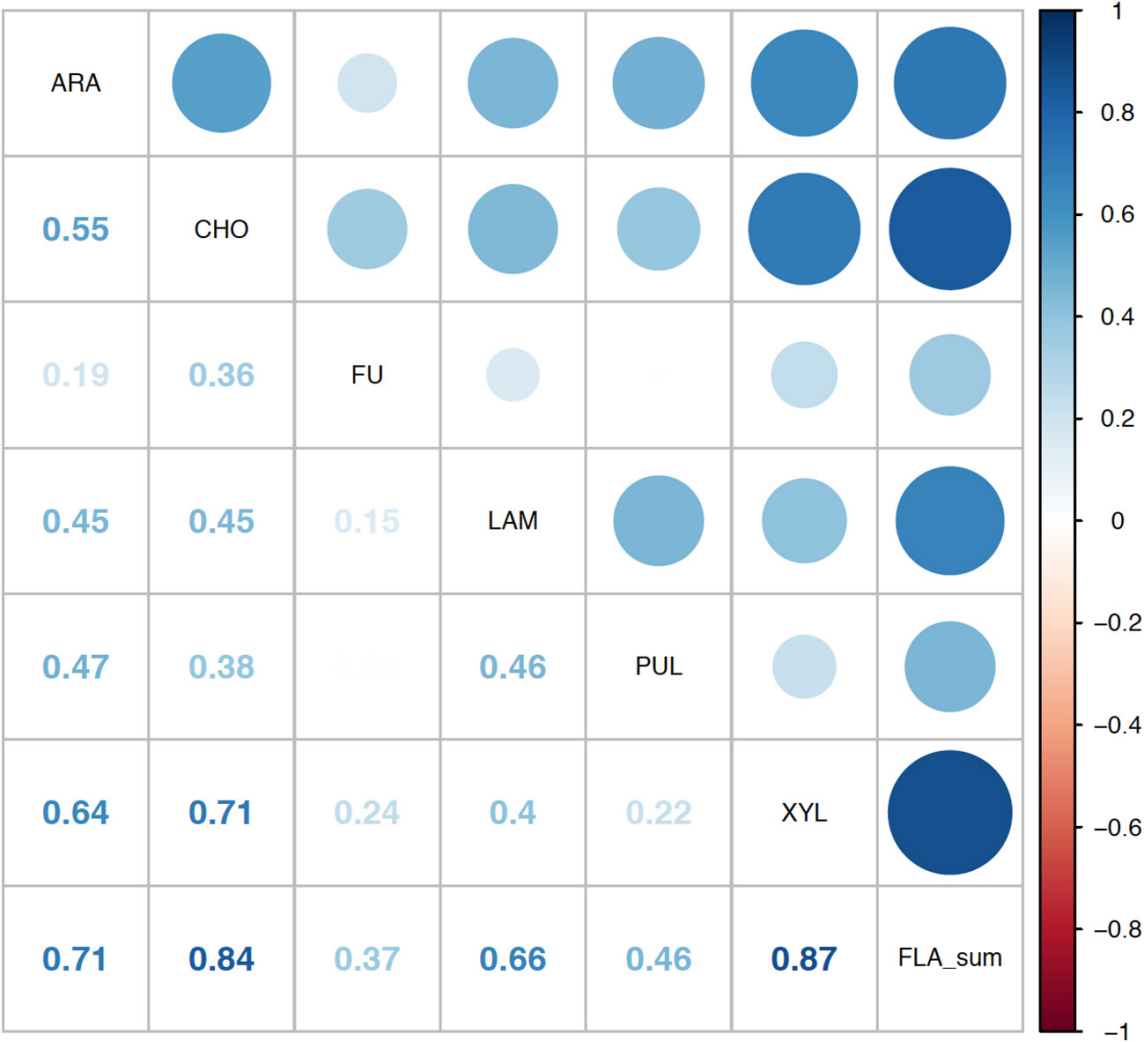
Correlation plot for the polysaccharide hydrolase activities contributing to the summed activity. ARA, arabinogalactan; CHON, chondroitin sulfate; FU, fucoidan; LAM, laminarin; PUL, pullulan; XYL, xylan; FLA_sum, summed polysaccharide hydrolase activities. Colors show intensity of correlations; numbers show corresponding correlation coefficients.

Correlation analysis of environmental parameters and microbial activities (Figure 10; *p*-values in Supplementary Table 6) showed some expected as well as unexpected correlations. Unsurprisingly, dissolved oxygen (DO) was strongly inversely correlated with temperature, and discharge, conductivity, and gage height also showed moderate correlation. Summed polysaccharide hydrolase activities (FLA; Figure 10) were positively correlated with temperature, and thus also inversely correlated with DO, although other enzyme activities did not show a notable correlation with temperature (or DO). Correlations among the other enzyme activities varied: β- and α-glu were strongly correlated with each other, and were more weakly correlated with Leu-MCA and phosphatase. Cell counts were inversely correlated with DOC, but leucine incorporation (bacterial protein production) showed only a strong inverse correlation with DO. Correlation analysis among the individual polysaccharide hydrolase activities (Figure 11; *p*-values in Supplementary Table 7) showed that summed activities were most strongly correlated with xylan and chondroitin hydrolysis, followed by arabinogalactan and laminarin hydrolysis.

### Effects of hurricane irene

Post-Hurricane Irene sampling (September 2011, plus August 2011 for Stns. T5 and T6) showed that phosphatase activities and DOC concentrations were considerably elevated compared to other sampling dates at the downriver stations (T5, T6, N6, N7; Table 2; Figures 3, 8). Although bacterial protein productivity was greatly elevated at Stn. N7 in September 2011 (Figure 4), the statistical significance of this measurement could not be calculated due to the small number of observations.

## Discussion

Microbial processing of organic matter in riverine systems can be influenced by a variety of physical, chemical, and biological factors that vary across a range of spatial and temporal scales (Singh et al., 2013). Most previous studies of microbially-driven carbon cycling in rivers have focused on either sampling a range of sites across a limited time period, or have investigated fewer sites over an annual cycle (e.g., Artigas et al., 2009; Tiquia, 2011; Frossard et al., 2012; Millar et al., 2015). In an effort to investigate some of the complexities of these interactions in rivers, we sampled both the Neuse and Tar-Pamlico Rivers across multiple seasons and sites. The range of Leu-MCA, β-glu, and phosphatase activities measured across sites and seasons in the Tar-Pamlico and Neuse Rivers proved to be similar to or slightly higher than rates measured across a broad range of riverine sites sampled at one season (e.g., Williams et al., 2012; Millar et al., 2015), as well as seasonal studies at fewer locations (Wilczek et al., 2005), suggesting that the Tar-Pamlico and Neuse Rivers are not atypical in their enzymatic activities. Cell counts were also generally similar to those reported from other riverine locations (Wilczek et al., 2005; Williams et al., 2012; Millar et al., 2015).

Two broad-scale spatial patterns in microbial enzyme activities emerged over the course of the study: higher peptidase activities at T6 compared to the other stations in the Tar-Pamlico River, and higher β-glucosidase and phosphatase activities in the Tar-Pamlico River compared to the Neuse River (Table 2; Figures 5, 6, 8). These spatial patterns in microbial enzyme activities contrast in particular with a lack of spatial patterns for microbial cell counts, and bacterial productivity that showed a different spatial pattern: upriver/downriver contrasts, and higher values at Stn. N7 than N1 (Table 2). Moreover, bacterial protein production correlated (inversely) only with dissolved oxygen, but not with any of the other activity measurements (Figure 10).

Microbial sources likely account for most of the enzymes active in the water column, but individual microbes can differ considerably in terms of activity, as exemplified by the differences in cell-count normalized bacterial production (Supplementary Table 2), which also demonstrated no distinct spatial patterns. Moreover, the capabilities of distinct members of microbial communities to carry out specific enzymatic function differs substantially, even among closely-related microbes (Xing et al., 2014). Since the measured enzyme activities are also an outcome of the kinetic characteristics of enzymes and their active lifetimes in the water column, as well as the quantity of enzymes produced, a lack of correlation between microbial cell numbers or bacterial productivity and enzyme activities is not entirely surprising. A lack of correlation between either cell counts or bacterial productivity and glucosidase and Leu-MCA activities has also been observed in other freshwater environments (e.g., Sieczko et al., 2015). The observation that summed polysaccharide hydrolase activities do not demonstrate the same spatial patterns seen for β-glucosidase activities—there is no difference between the activities measured in the Tar-Pamlico and in the Neuse River (Table 2)—is likely due to the fact that the overall ability to produce specific extracellular enzymes is non-uniformly distributed among members of microbial communities (Zimmerman et al., 2013). Furthermore, the longer incubation times for the polysaccharide hydrolase measurements (3 days, compared to hours for the β-glucosidase activities) allows time for growth and induction responses to polysaccharide addition, which may have masked any initial differences among sites.

Given prior observations of a limited spectrum of polysaccharide-hydrolyzing enzyme activities in aquatic systems (e.g., Ziervogel and Arnosti, 2009; Arnosti et al., 2011; Ziervogel et al., 2014), the hydrolysis of all six polysaccharide substrates at every station in the Neuse and Tar-Pamlico Rivers at timepoints throughout the year was remarkable (Supplementary Table 3). This breadth of hydrolytic capabilities has seldom been observed in other locations; even nutrient addition has not led to hydrolysis of some polysaccharides in some locations (Steen and Arnosti, 2014). Typically, over time-courses of incubations lasting well over 3 days, only a subset of polysaccharides was hydrolyzed (e.g., Steen et al., 2008; Arnosti et al., 2011; Ziervogel et al., 2014). Moreover, this broad range of hydrolytic capabilities in the Neuse and Tar-Pamlico Rivers was observed at timepoints when summed hydrolysis rates were low, as well as at times when summed hydrolysis rates were high, as at Stn. N1 in February and June 2012, when summed hydrolysis rates were 6 and 65 nmol monomer L^−1^ h^−1^, respectively (Figure 9; Supplementary Table 3).

The broad hydrolytic capabilities of microbial communities in the Neuse and Tar-Pamlico Rivers may be due to the extensive and diverse watersheds of both rivers, as well as the occurrence of seasonal flooding events, leading to substantial terrestrial input into the systems, which provides the microbial community with a greater quantity and diversity of organic matter sources. In particular, the lower reaches of both rivers are subject to frequent overbank flooding because there are no large dams to control flooding and because these low-elevation coastal plain rivers have low banks that help facilitate frequent flooding (Peng et al., 2004; Reed et al., 2008). Although previous studies in other riverine systems have suggested that freshwater and estuarine organic matter of autochthonous, as opposed to allochthonous, origin is of greater importance to the microbial community for uptake (McCallister et al., 2006), significant input of terrestrial organic matter may well influence, and possibly increase, hydrolytic capabilities. Such a broad range of DOM sources may also account for the observation that a site within Pamlico Sound also showed hydrolysis of all six polysaccharide substrates, in contrast to a nearby site on the continental shelf, where only four of the substrates were hydrolyzed (D'Ambrosio et al., 2014). Throughout all seasons, the comparatively high contributions of xylan hydrolysis to summed hydrolysis rates may be an additional indication of the importance of terrestrial sources of organic matter to both rivers, since xylan is a major constituent of land plants (Ebringerova and Heinze, 2000), as well as some algae. High xylan hydrolysis rates have also been measured in the Chesapeake Bay (Steen et al., 2008) and Delaware River (Ziervogel and Arnosti, 2009). Comparably high contributions of both chondroitin and xylan to summed hydrolysis rates have to date been observed in the Delaware River (Ziervogel and Arnosti, 2009), and in the Gulf of Mexico, at sites that presumably also have the potential to be influenced by terrigenous input from the Mississippi River (Arnosti et al., 2011; Steen et al., 2012).

Spatial differences in DOC concentrations were evident—most notably elevated downstream DOC concentrations, particularly at Stn. N7—but this pattern is not reflected in most of the enzyme activities measured in the two rivers (Table 2). The elevated DOC concentration particularly at Stn. N7 may in part reflect lateral input at the downstream locations, as well as the hydrologic disconnect shown by discharge and gage height (Figure 2; Supplementary Figure 1) of upstream and downstream stations. DOC contributed via different hydrologic flow paths can differ considerably in source as well as composition (Singh et al., 2013). Moreover, lateral input of DOC may include comparatively more microbially recalcitrant organic matter that survives photochemical and microbiological processing, and does not enhance bacterial activity. A lack of clear relationships between DOC and enzyme activities was also reported for the large tributaries of the lower Mississippi River, where site-to-site differences for a single river were as large as between-river variations in enzyme activities (Millar et al., 2015). A similar lack of relationship between the origin and optical properties of DOM and glucosidase or Leu-MCA activities was reported for the Danube floodplain (Sieczko et al., 2015). The Danube floodplain sites also showed little systematic variation in bacterial abundance or productivity, despite differences in glucosidase and peptidase activities (Sieczko et al., 2015).

The lack of broad seasonal trends in microbial activities and abundance was something of a surprise. Although glucosidase, peptidase, and phosphatase activities were lowest in Jan/Feb (Table 2), when temperatures were also lowest (Supplementary Table 1), low activities were at times also measured in other months—for example, in June—when seasonal temperatures were near or at their maximum. Across the entire study period, temperature correlated with summed polysaccharide hydrolase activities (Table 2), but summed activities were sometimes low at times when water temperatures were high (e.g., June 2011 at Stn. N7; Figure 9) Other studies have shown seasonality to be an important factor associated with changes in microbial community activities (Artigas et al., 2009), with temperature as an important controlling variable (Wilczek et al., 2005). A high-resolution investigation of enzyme activities at a single site in the coastal Pacific, however, demonstrated that substantial variations in enzyme activities occurred on timescales shorter than 1 month, and that seasonal patterns were not clearly evident (Allison et al., 2012).

An investigation of microbial community composition along the salinity gradient of the Columbia River and its estuary, extending into the coastal ocean, also found that strong spatial patterns overwhelmed seasonal patterns, which were more evident within individual groups of bacteria (Fortunato et al., 2012). Metabolic potential, as represented by metagenomes, varied comparatively little along this same gradient, but metatranscriptomic data showed considerable variability that was unrelated to season or salinity (Fortunato and Crump, 2015), suggesting that the communities were reacting to localized environmental conditions. In the case of the Tar-Pamlico and Neuse Rivers, factors that are unrelated to season—such as land use, and abundance of natural land cover (Table 1)—may correlate more strongly with enzyme activities (Williams et al., 2012). The interplay between factors that do and do not have consistent seasonal trends may thus help drive enzymatic activities, and obscure seasonal correlations. However, evidence suggests that microbial communities in freshwater, estuarine, and marine systems are able to respond rapidly to increased organic matter inputs (Williams and Jochem, 2006; Allison et al., 2012). Such rapid responses may be the reason that consistent, long-term seasonality is not evident in the Neuse and Tar-Pamlico Rivers, as microbial communities may respond to factors that change on timescales different than the length of time between sampling dates.

The passage of Hurricane Irene across the eastern half of North Carolina in August 2011, however, is an example of an event that left a signature discernable in the lower reaches of the Tar-Pamlico and Neuse Rivers even two-plus weeks post-event. Phosphatase activities, DOC concentrations, and cell counts showed significant differences in September 2011 (and August, for Stns. T5 and T6) compared to other sampling months at these sites (Table 2). (Note that the August sampling was a day after Hurricane Irene passed through North Carolina; the September samplings were ca. 2 weeks post-hurricane). DOC concentrations at Stns. T5, T6, and N7 all were far higher in September 2011 than in any other month at the same location (Figure 3), despite the probability that the concentrations measured 2 weeks post-event were lower than the maximum concentrations. Maximal DOC input into the Neuse estuary lagged maximum discharge by ~1 week (Brown et al., 2014), presumably due to lateral inputs into the upper reaches of the Neuse River in response to extensive flooding. Flooding and elevated discharge were likely responsible for changes in microbial community composition in the Tar-Pamlico River, where microbial community composition downstream (Stn. T6) shifted considerably post-hurricane (Balmonte et al., 2016). Prior to the hurricane, the community composition of Stns. T1 and T6 were notably dissimilar. Immediately post-hurricane (August 2011) as well as 2 weeks later (Sept. 2011), there was evidence of coupling between upstream and downstream stations, as well as post-hurricane microbial input from terrestrial sources. These distinct microbial signatures were less evident by November 2011 (Balmonte et al., 2016).

Although DOC concentrations measured in Sept. 2011 were similar in the Neuse and Tar-Pamlico Rivers (Figure 3), the composition of this DOC was likely different, given the difference in watersheds (Figure 1; Table 1) and the notable differences in responses of the microbial communities in the two rivers to this DOC. Bacterial production on a cell-specific basis reached a maximum in Sept. 2011 at Stn. N7 more than an order of magnitude higher than otherwise measured at this station, and more than four times greater than at Stn. T6 at the same time (Supplementary Table 2); bacterial production at Stn. N7 was also maximal at this station on volume-specific basis (Figure 4). Phosphatase activities were also greatly elevated at Stn. N7, but not at Stn. T6 (Figure 8). In the Tar-Pamlico River, by contrast, bacterial production, glucosidase, and Leu-MCA activities at Stn. T6 were not notably elevated even during the August 2011 sampling (Figures 5–7), the day after the passage of Hurricane Irene. Although no data on the chemical characteristics of Hurricane-Irene associated DOC are available from the Tar-Pamlico and Neuse Rivers, Hurricane Irene-associated water collected within a Maryland watershed showed distinct spectroscopic characteristics compared to water collected at other times, likely due to differences in sources and flow-paths (Singh et al., 2013). The differences in land use, drainage, and flow paths thus may have led to considerable compositional differences in the DOC added to the Neuse and Tar-Pamlico Rivers as a consequence of Hurricane Irene. Together, these data suggest that there was a microbial response to the DOC added to the Neuse River, but not the Tar-Pamlico River, post Hurricane Irene, but this response did not involve the glucosidase, Leu-MCA, or polysaccharide hydrolase enzymes whose activities we measured, or (alternatively), any enzymatic response in the Neuse River was shorter-lived than the elevation of DOC concentration, two-plus weeks post-event.

Complex trends of organic carbon remineralization characterize microbial activities in the Tar-Pamlico and Neuse River systems. Broad-scale spatial patterns—in particular, higher β-glucosidase and phosphatase activities in the Tar-Pamlico compared to the Neuse River, as well as higher downstream Leu-MCA activities in the Tar-Pamlico River—are evident in this study, but no single factor can be pinpoint as the most influential in shaping community activities; even large-scale events such as a hurricane's passage elicited different responses in the two rivers. Future studies of similar spatiotemporal scales, ideally including focused investigation of DOC characteristics and flow paths, will be necessary for clearer understanding of the factors that drive microbial community activities and organic matter remineralization across aquatic gradients.

## Author contributions

CA and BM designed the study. AB, KZ, SG, and SS collected the samples, carried out the incubations, and collected the data. AB, KZ, SG, SS, and CA analyzed the data. AB and CA wrote the manuscript, with input from all co-authors. We are very grateful to the two reviewers, whose thoughtful comments considerably improved the manuscript.

### Conflict of interest statement

The authors declare that the research was conducted in the absence of any commercial or financial relationships that could be construed as a potential conflict of interest.

## Supplementary material

Supplementary Figure 1 Gage height (ft) for the Tar-Pamlico and Neuse rivers. Solid circle show time points at which were collected; lines connect consecutive sampling dates.

Supplementary Table 1 Temperature, salinity, dissolved oxygen, and pH at the date and time of sampling for all stations and sites.

Supplementary Table 2 Leucine incorporation, cell counts, leucine incorporation per cell, and DOC concentration for all stations and sites.

Supplementary Table 3 Polysaccharide hydrolase activities (nmol monomer L^−1^ h^−1^) for all stations and sites. Ara, arabinogalactan; cho, chondroitin sulfate; fu, fucoidan; lam, laminarin; pul, pullulan; xyl, xylan.

Supplementary Table 4 Gage height and discharge on the date of sampling for each station and date.

Supplementary Table 5 α-glucosidase, β-glucosidase, and leu-aminopeptidase activities (nmol L^−1^ h^−1^) for each station and date.

Supplementary Table 6 Data shown in Figure 10; *P*-values as calculated using the corrplot package in R. Values where *P* < 0.05 are shown in bold font.

Supplementary Table 7 Data shown in Figure 11. *P*-values as calculated using the corrplot package in R. Values where *P* < 0.05 are shown in bold font.
