# Supplementary figures and images for: A Multi-season Investigation of Microbial Extracellular Enzyme Activities in Two Temperate Coastal North Carolina Rivers: Evidence of Spatial but Not Seasonal Patterns
Source: Front Microbiol. 2017 Dec 22;8:2589. doi: 10.3389/fmicb.2017.02589 (PMC5743733; doi:10.3389/fmicb.2017.02589)

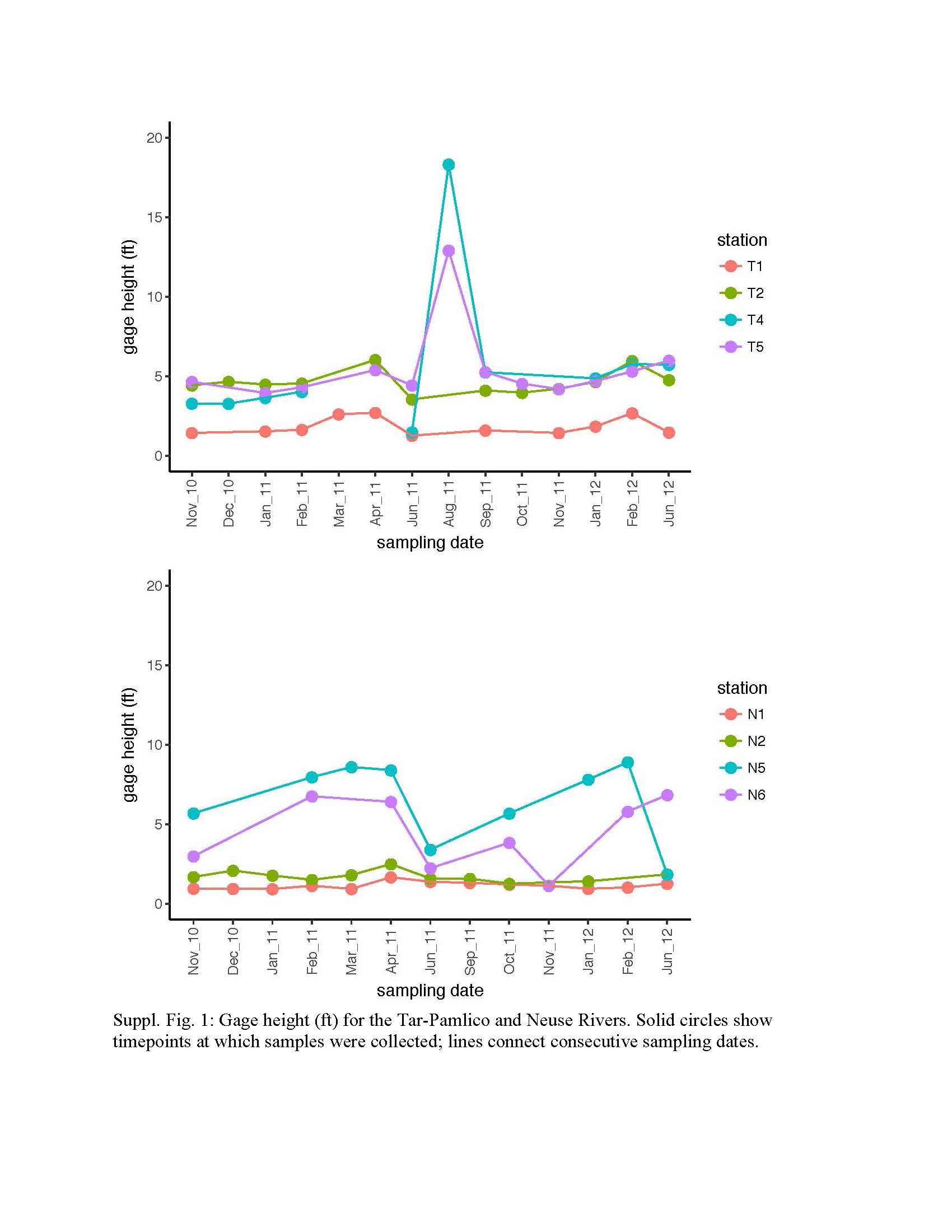

Supplement: Supplementary Figure 1 — Gage height (ft) for the Tar-Pamlico and Neuse rivers. Solid circle show time points at which were collected; lines connect consecutive sampling dates. [file Image1.JPEG]
